# Evaluation of Three Commercial PCR Assays for the Detection of Azole-Resistant *Aspergillus fumigatus* from Respiratory Samples of Immunocompromised Patients

**DOI:** 10.3390/jof7020132

**Published:** 2021-02-11

**Authors:** Ulrike Scharmann, Lisa Kirchhoff, Andrea Hain, Jan Buer, Michael Koldehoff, Joerg Steinmann, Peter-Michael Rath

**Affiliations:** 1 Institute of Medical Microbiology, University Hospital Essen, University of Duisburg-Essen, 45147 Essen, Germany; lisa.kirchhoff@uk-essen.de (L.K.); andrea.hain@uk-essen.de (A.H.); jan.buer@uk-essen.de (J.B.); joerg.steinmann@klinikum-nuernberg.de (J.S.); peter-michael.rath@uk-essen.de (P.-M.R.); 2 Department of Hematology and Stem Cell Transplantation, West German Cancer Centre, University Hospital Essen, University of Duisburg-Essen, 45147 Essen, Germany; michael.koldehoff@uk-essen.de; 3 Institute of Clinical Hygiene, Medical Microbiology and Infectiology, Klinikum Nürnberg, Paracelsus Medical University, 90419 Nuremberg, Germany

**Keywords:** *Aspergillus*, invasive fungal infection, azole-resistance, invasive pulmonary aspergillosis

## Abstract

This is the first study comparing three commercially available PCR assays for the detection of *Aspergillus* DNA from respiratory specimen of immunocompromised patients and the presence of *cyp51A* gene mutations. Bronchoalveolar lavages (BALs, *N* = 103) from patients with haematological/oncological underlying diseases were retrospectively investigated. The performance of three PCR assays, namely MycoGENIE^®^ *Aspergillus fumigatus* Real-Time PCR Kit (Adamtech), Fungiplex^®^ *Aspergillus* Azole-R IVD Real-Time PCR Kit (Bruker Daltonik GmbH) and AsperGenius^®^ (PathoNostics B.V.), were evaluated. All patients were categorised following current EORTC/MSG criteria, with exclusion of the PCR-results. From the 11 invasive pulmonary aspergillosis (IPA) probable samples, eight were detected with MycoGENIE^®^, resulting in a sensitivity of 80% and a specificity of 73%. Furthermore, Fungiplex^®^ resulted in six positive BALs with a sensitivity of 60% and a specificity of 91% and AsperGenius^®^ in seven positive BAL samples, with a sensitivity of 64% and a specificity of 97%. No proven IPA was detected. One isolate showed phenotypically an azole-resistance, which was also detected in each of the tested PCR assays with the mutation in TR34. The here tested PCR assays were capable of reliably detecting *A. fumigatus* DNA, as well as differentiation of the common *cyp51A* gene mutations. However, evaluation on the AsperGenius^®^ assay revealed a low risk of false positive results.

## 1. Introduction

Invasive pulmonary aspergillosis (IPA) mostly occurs in immunocompromised patients, especially in patients with haematological, solid organ malignancies or after allogeneic and solid organ transplantation [1,2,3,4]. However, occurrence of invasive fungal infections increased in non-immunocompromised patients with other pulmonary diseases, such as influenza or COVID-19 infection [5,6].

Microbiological detection of *Aspergillus fumigatus*, as the representative mould for IPA, is still problematic [7,8,9]. In contrast to culturing methods, biomarkers such as galactomannan (GM) are a commonly applied tool, being more sensitive in diagnosing an IPA [10]. While biomarkers are not specific for the identification of the species of fungi, the combination of biomarkers, such as GM and (1-3)-β-D-glucan (BDG), with PCR assays enables an even more reliable diagnosis of an IPA [11,12,13].

As first-line antifungal therapy, voriconazole (VOR) or isavuconazole are recommended by European guidelines [10]. Antifungal susceptibility testing raised in importance as intrinsic and acquired resistances against triazoles among *Aspergillus* strains are increasingly recognised [14,15]. For susceptibility testing of *A. fumigatus* isolates, broth microdilution is still the gold standard test, and, for azole-resistance detection, screening in four-well azole-agar plates is recommended [10,16]. However, for these methods, culture-based identification is necessary. Since culturing of *Aspergillus* from respiratory samples is only rarely successful, empiric antifungal therapy is commonly the routine. While azole-resistance is associated with treatment failures and thus with a higher mortality [17,18], a fast and reliable diagnosis of an IPA and detection of possible azole-resistance is of high importance.

Recently, EORTC/MSG criteria for definition of proven or probable invasive fungal infection were updated by adding the PCR as a diagnostically criterion [19]. Different PCR assays are commercially available for the detection of *Aspergillus* specific DNA and additionally the detection of *cyp51A* gene mutations, which are associated with azole-resistance, directly from respiratory material. This is the first study comparing three commercially available PCR assays for the detection of *Aspergillus* DNA and the mutation associated with azole-resistance directly from respiratory samples of immunocompromised patients.

## 2. Materials and Methods

### 2.1. Patients and Specimens

In total, 103 bronchoalveolar lavages (BALs) from 93 immunocompromised patients with a high risk of IPA, treated at the Department of Hematology and Stem Cell Transplantation, University Hospital Essen, Germany, were included in this study. Samples were collected between 2017 and 2019 at the University Hospital in Essen, Germany.

Patients were classified in accordance with the EORTC/MSG criteria to define an IPA, excluding PCR results [19].

The study was approved by the local ethics committee (ethics committee of the Faculty of Medicine Essen, University of Duisburg-Essen, Essen, Germany; reference number 18-8376-BO, 17 October 2018).

### 2.2. Conventional Diagnostic

All clinical samples were analysed after conventional microbiological diagnostic tests had been performed. Routine mycological diagnostic tests consisted of culturing on malt agar (Oxoid, Basingstoke, UK) for seven days at 30 °C. In the case of fungal growth, susceptibility testing was performed by ETEST^®^ for VOR (bioMérieux, Marcy-l′Étoile, France) and itraconazole (Liofilchem, Roseto degli Abruzzi, Italy). Minimal inhibitory concentrations were determined in accordance with EUCAST guidelines version 10.0 [20]. Furthermore, GM index was determined via Platelia *Aspergillus* EIA (BioRad, Marnes-la-Coquette, France) from BAL and serum samples, following the manufacturer’s instructions. Results from BAL samples with a GM index ≥1 and from serum samples with a GM index >0.5 were interpreted as positive.

### 2.3. DNA Extraction

For DNA extraction, 500 µL of respiratory sample were used. Extraction was performed in a Maxwell16 instrument (Promega GmbH, Walldorf, Germany) with the Maxwell16 Tissue LEV Total DNA/RNA Purification Kit (Promega GmbH, Walldorf, Germany) according to the recommendations of the manufacturer. The final extraction volume was 100 µL.

### 2.4. Real-Time PCR Assays

The performance of three PCR kits, MycoGENIE^®^ *Aspergillus fumigatus* Real-Time PCR Kit (Adamtech, Pessac, France), Fungiplex^®^ *Aspergillus* Azole-R IVD Real-Time PCR Kit (Bruker Daltonik GmbH, Bremen, Germany) and AsperGenius^®^ (PathoNostics B.V., Maastricht, The Netherlands), was investigated. Assays were carried out following manufacturer’s instructions. Each assay was performed in a RotorGeneQ thermocycler (Qiagen, Hilden, Germany). Positive and negative controls were performed in each assay.

The target of the MycoGENIE^®^ assay is *A. fumigatus* 28S rRNA gene and the mutation TR34/L98H for the detection of an azole-resistance.

The Fungiplex^®^ PCR assay aims to detect *Aspergillus* spp. and the target for resistance gene are the mutations TR34 and TR46 in the *cyp51A* gene, while AsperGenius^®^ PCR assay identifies *Aspergillus* spp., *A. terreus* and *A. fumigatus* (28S rRNA gene). Different molecular azole-resistance mutations as TR34, L98H, T289A and Y121F on single copy *cyp51A* gene could be detected with AsperGenius^®^ PCR assay.

PCR results were categorised as positive if cycle threshold (Ct) values of DNA detection of *A. fumigatus/Aspergillus* spp. and an azole-resistance associated mutation were lower than the respective cut-off (MycoGENIE^®^ Ct < 40, Fungiplex^®^ Ct < 45, and AsperGenius^®^ Ct < 36).

Analyses were done according to Good Clinical Practice guidelines.

### 2.5. Statistical Analysis

PCR results were compared to conventional diagnostic tools (culture, susceptibility testing, GM index in BAL and serum) and patients’ clinical conditions. All patients were categorised after EORTC/MSG criteria, while the PCR results were excluded regarding the evaluation process. Patients were categorised in the probable IPA group if all three criteria (host factors, clinical features and mycological evidence) were fulfilled as defined by Donnelly et al. [19]. The possible IPA group was defined if one of the named criteria was not fulfilled. Not categorisable patients missed two of the three named criteria. Not categorisable patients were classified according to the algorithm for the intensive care unit patients, as described by Blot et al. [21].

Positive (PPV) and negative predictive values (NPV), sensitivity and specificity were calculated according to the following definitions. Correct positive was defined if *Aspergillus* DNA was detected in the PCR assay in the group of probable IPA patients. False positive was defined if the patient was categorised in the probable IPA group or was not categorisable, but *Aspergillus* DNA could be detected in the PCR assay. Correct negative was defined if no *Aspergillus* DNA could be detected in the possible or in the not categorisable group. False negative was defined if no *Aspergillus* DNA could be detected in the probable IPA group.

## 3. Results

One hundred and three BAL samples from 93 patients were investigated in this study. Patient’s details and IPA-categorisations are summarised in Table 1. According to the EORTC/MSG criteria, 11 samples from 10 different patients were categorised as probable IPA, 51 samples from 46 patients as possible IPA and 41 samples from 37 patients did not fulfill the criteria. No proven IPA could be detected. From the 41 not-categorisable patients, all patients could be classified in the group of respiratory tract colonisation due to the algorithm of intensive care unit patients, as described by Blot et al. [21].

Overall, 44 patients received antifungal therapy/prophylaxis while BAL was obtained. Regarding azoles, 18 patients received VOR, 8 posaconazole and 4 fluconazole. In three cases, caspofungin and in 11 cases liposomal amphotericin was administered. No influences on PCR results were observed. No combination therapy was used. As one of the EORTC criteria, GM was investigated in 98 BAL and 62 serum samples.

The microbiological results from samples of the patients of the probable IPA group are shown in Table 2.

**Figure 1:**
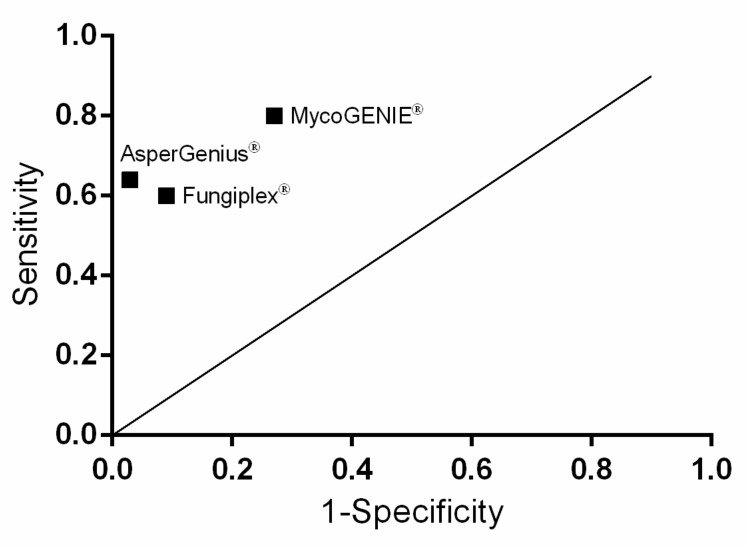
Clinical sensitivity and specificity for three PCR assays. AsperGenius^®^ shows the best connection between sensitivity and specificity, while MycoGENIE^®^ shows a high sensitivity but low specificity. Diagonal line represents random results.

Using molecular techniques, 35 samples were detected to be positive (31 haemato-oncological patients and four with a solid organ tumour) in at least one of the included PCR assays. For the EORTC/MSG IPA probable group, MycoGENIE^®^ resulted positive in eight BAL specimens, resulting in a sensitivity of 80% and a specificity of 73%. Furthermore, Fungiplex^®^ resulted in six positive BAL with a sensitivity of 60% and a specificity of 91% and AsperGenius^®^ in seven positive BAL samples with a sensitivity of 64% and a specificity of 97%. Statistical analysis is depicted in Figure 1, showing the data based on random results. Positive (PPV) and negative predictive values (NPV) are depicted in Table 3 and Table 4.

Out of the eleven probable categorised samples, two samples were positive in culture (25%). One of these two samples showed phenotypically an azole-resistance, which was also detected in each of the tested PCR assays with the mutation in TR34. The molecular detection was obtained directly from the BAL, the same material of which *A. fumigatus* was cultured and phenotypically resistance was detected.

## 4. Discussion

While the conventional culturing of *A. fumigatus* from respiratory samples is linked to a low detection rate, biomarker- and molecular-based methods become an important tool for the detection of *Aspergillus* [22]. PCR results were integrated in the update of classification criteria of EORTC/MSG for IPA identification [19]. Since azoles are recommended as first line therapy for patients suffering an IPA, not only the identification of *Aspergillus* but also the identification of azole-resistance has high clinical relevance [10]. Different studies identified numerous mutations in the *cyp51A* gene, which are responsible for resistances against triazoles, resulting in therapy failures [17,18,23,24]. This is the first study evaluating three different commercially available PCR assays regarding their performance on detection of *A. fumigatus* DNA and corresponding mutations, enabling an identification of azole-resistance *Aspergillus* directly from respiratory samples of immunocompromised patients.

With the MycoGENIE^®^ assay, we detected the highest sensitivity of 80% but the lowest specificity (73.2%) and PPV (26.7%), when investigating the probable IPA group for *Aspergillus* DNA. Different study groups evaluated MycoGENIE^®^ assay and revealed a sensitivity of 40%, 71% and 92.9% and a specificity of 69%, 90.1% and 100% when investigating proven and probable IPA patients [25,26,27]. One of these study groups showed a positive effect with statistical relevance when adapting the interpretation criteria [25]. In this study, they investigated 123 BAL samples from 114 patients with stem cell transplantation and/or haematological malignancies with definition of 2 proven and 28 probable IPA patients [25]. When Mikulska et al. added the PCR or GM results to the criteria, the sensitivity rose significant (from 40% to 83%), while specificity was unchanged [25]. Sensitivity also increased (from 40% to 61%) in the group of patients who received antifungal therapy, while specificity was nearly unchanged (69% vs. 64%) [25]. In our samples, a positive GM value (≥1) in BAL samples was detected for any as probable IPA classified sample; thus, according to Mikulska et al. [25], sensitivity was quite high (80%). In contrast, PPV was 26.7%, so most of the positive PCR results were not reliable.

With the Fungiplex^®^ PCR assay, we reached a higher PPV of 42.9, while other statistical values were comparable to AsperGenius^®^ assay. This is the first study evaluating the Fungiplex^®^ assay with clinical BAL samples. The assay reached a high specificity and a high NPV independent from antifungal therapy or prophylaxis.

AsperGenius^®^ seems to be a reliable commercially available kit for rapid and secure detection of IPA (Figure 1). Here, the use of AsperGenius^®^ showed a low risk of false positive IPA results. At the same time, probable IPAs were detected sufficiently.

In a previous study, our group tested the performance of AsperGenius^®^ on 100 consecutive allogenic haematopoietic stem cell transplant recipients [28]. Samples were collected from 2015 to 2017, and 23 probable cases were identified [28]. Sensitivity was 65%, specificity and PPV 100% and NPV 91% for probable IPA group [28]. Other study groups detected sensitivity about 84% and specificity between 80% and 91.4%, PPV between 76% and 84% and NPV between 87% and 94.6%, respectively [18,29].

Differing numbers of the sensitivity and specificity between different studies might occur due to different requirements, such as composition of patient cohort or criteria for categorisation of the patients in different groups. This contributes to a more complicated comparison of PCR assays between different study groups. Furthermore, a lower analytical sensitivity can reveal in the case of non-*fumigatus Aspergillus* species as cause of an IPA. Therefore, the use of a species-specific assay will lead to false negative results as these often reveal a lower accuracy and sensitivity [30]. Furthermore, only small numbers of positive results could bias the statistical analysis. Even though contamination by inhaled conidia is unlikely, it cannot be fully ruled out.

There are two approaches available: genus-specific PCR assays and species-specific PCR assays. Here, both types were included with Fungiplex^®^ being genus-specific and MycoGENIE^®^ being species-specific. In contrast, AsperGenius^®^ is detecting *A. fumigatus* and *A. terreus* on a species level, while all other non-*fumigatus Aspergillus* species are labelled as *Aspergillus* spp. on genus level. In a study from Morton et al., the use of genus-specific PCR assays for IPA identification rather than species-specific PCRs has been recommended [30].

It has been shown that *Aspergillus* isolates with an azole-resistance were linked to an increased risk of therapy failure [18,31]. In a prospective multicentre international surveillance study, the prevalence of azole-resistance of *A. fumigatus* isolates in Europe was detected to be 3.2% with TR34/L98H mutation as the most frequently detected mutation (50%) [32]. We found only one azole-resistant *A. fumigatus* isolate, which was successfully detected in each of the PCR assays. Regarding the detection of mutations in the *cyp51A* gene, MycoGENIE^®^ and AsperGenius^®^ assay worked reliably in various studies [25,27,28,29]. Furthermore, the detection of mixed infections with azole-resistant and susceptible *A. fumigatus* isolates was possible with AsperGenius^®^, while cultures were negative [33]. However, considering the number of false negative results, azole-resistance of *A. fumigatus* isolates could be underestimated.

In conclusion, we found no influence of antifungal therapy or prophylaxis on any of the PCR results. The MycoGENIE^®^ assay produced results with the highest sensitivity, whereas Fungiplex^®^ and AsperGenius^®^ assays produced results with the highest degree of specificity. BAL samples obtained in the hospital’s bone marrow transplant unit from patients with haematological/oncological conditions are routinely screened using PCR and mycological biomarker assays. Therefore, for our patient cohort, the higher specificity provided by the AsperGenius^®^ assay is most valuable in reliable diagnosis of an IPA. One more advantage of AsperGenius^®^ is that melting curve analysis for detection of azole-resistance mutation enables the user to detect mixed infections with both wild type isolates and isolates harbouring a mutation in *cyp51A* gene [33]. Furthermore, next to the above-named mutation, there are several other known mutations which are not detected when using MycoGENIE^®^ and Fungiplex^®^ assay [31].

Taking everything into account, we would recommend the AsperGenius^®^ PCR assay for the detection of *Aspergillus* DNA and the corresponding mutation in one of the tested mutations of the *cyp51A* gene to rule out an IPA when investigating respiratory material.

The limitations of this study are that only patients from one centre were integrated. Additionally, no BAL sample from a patient with a proven IPA and only a small number of patients with a probable IPA could be investigated. The small number of positive results could bias the final interpretation of the data. Furthermore, only one of the included isolates showed an azole-resistant phenotype.

## Figures and Tables

**Table 1:** Characteristics of patient cohort.

**Gender [n (%)]**			**Male**	67 (72.0)
			**Female**	26 (28.0)
**Age [years]**			Median	61
			Min.	18
			Max.	92
**Underlying condition [n (%)]**				
**Haematological malignancy**				**70 (75.3)**
Acute leukemias				
• AML				25 (26.9)
• ALL				7 (7.5)
MDS				8 (8.6)
Chronic leukemias				
• CLL				1 (1.1)
• MM				7 (7.5)
Myeloproliferative diseases				
• CML				6 (6.5)
• MPS				6 (6.5)
Lymphoma				
• HD				2 (2.2)
• NHL				6 (6.5)
Aplastic anaemia				2 (2.2)
Allogenic stem cell transplantation [n (% of haematological malignancy)]				41 (58.6)
**Solid Cancer**				**20 (21.5)**
Lung (SCLC/NSCLC)				7 (7.5)
Intestine				5 (5.4)
Mouth				4 (4.3)
Bladder				1 (1.1)
Sarcoma				1 (1.1)
Testis				1 (1.1)
Breast				1 (1.1)
**Organ transplantation**				**1 (1.1)**
Kidney				1 (1.1)
**Other**				**2 (2.2)**
**EORTC/MSG**	**Probable**	**Possible**	**Not categorisable**	**Total**
Samples [n]	11	51	41	103
Patients [n]	10	46	37	93

Abbreviations: AML, acute myeloma leukaemia; ALL, acute lymphatic leukaemia; MDS, myelodysplastic syndrome; CLL, chronic lymphocytic leukaemia; MM, multiple myeloma; CML, chronic myeloid leukaemia; MPS, myeloproliferative syndromes; HD, Hodgkin’s lymphoma; NHL, non-Hodgkin lymphoma; SCLC, Small Cell Lung Cancer; NSCLC, Non-Small Cell Lung Cancer.

**Table 2:** Characteristics of samples from patients categorised in the probable IPA group.

Patient ID	MycoGENIE^®^	Fungiplex^®^	AsperGenius^®^	Culture	GM BAL	GM Serum
**3**	pos	pos	pos	*A. fumigatus*	5.6	0.5
**5**	inhibited	inhibited	neg	neg	4.4	nd
**11**	pos	neg	neg	neg	3.6	neg
**12**	neg	neg	neg	neg	1.8	1.3
**12**	pos	pos	pos	neg	1.0	neg
**19**	pos	pos	pos	neg	4.7	neg
**31**	pos	pos	pos	*A. fumigatus*	4.1	nd
**36**	pos	pos	pos	neg	4.3	neg
**47**	pos	neg	pos	neg	2.1	nd
**78**	neg	neg	neg	neg	3.9	neg
**82**	pos	pos	pos	neg	3.0	neg

Abbreviations: GM, galactomannan; BAL, bronchoalveolar lavage; pos, positive; neg, negative; nd, not done.

**Table 3:** Positive PCR assays categorised as probable, possible and not categorisable IPA (EORTC/MSG).

	MycoGENIE^®^		Fungiplex^®^		AsperGenius^®^	
	*Af*	TR34/L98H	*Aspergillus* spp.	TR34/TR46	*Af*/*Aspergillus* spp.	TR34/l98H/T289A/Y121F
**Probable IPA (*N* = 11)**	8	1 (TR34/L98H)	6	1 (TR34)	4/3	1 (TR34/L89H)
**Inhibited PCR results**	1		1		0	
**Possible IPA (*N* = 51)**	13	0	7	0	1/1	0
**Not categorisable IPA (*N* = 41)**	9	0	1	0	0/1	0
**Total (*N* = 103)**	30	1	14	1	5/5	1

Abbreviations: Af, *Aspergillus fumigatus*; IPA, invasive pulmonary aspergillosis.

**Table 4:** Statistical analysis of samples categorised as probable IPA vs. possible/not categorisable IPA (EORTC/MSG).

	MycoGENIE^®^	Fungiplex^®^	AsperGenius^®^
**PPV**	0.267	0.429	0.700
**NPV**	0.968	0.954	0.957
**Sensitivity**	0.8	0.6	0.636
**Specificity**	0.732	0.912	0.967

Abbreviations: PPV, positive predictive value; NPV, negative predictive value.

## Data Availability

The data presented in this study are available on request from the corresponding author. The data are not publicly available due to ethical restrictions.
